# Supplementary material for: Effect of Biopesticide Novochizol on Development of Stem Rust Puccinia graminis f. sp. tritici in Wheat, T. aestivum L
Source: Plants (Basel). 2024 Dec 9;13(23):3455. doi: 10.3390/plants13233455 (PMC11644718; doi:10.3390/plants13233455)
Supplement: Supplementary file 1 [file plants-13-03455-s001.zip › plants-3336380-supplementary.pdf]

Table S1 Summary of the significant difference (p-values) obtained with Mann-Whitney U Test. At p-values <0,05 – significant difference between the compared groups, p-values >0,05 – not significant difference. Designations as in Fig. 2.

**AA**	C							**TPC**	C						
	2 h	7 h	10 h	24 h	72 h	144 h	240 h		2 h	7 h	10 h	24 h	72 h	144 h	240 h
N	<0,05	<0,05	<0,05	<0,05	>0,05	>0,05	<0,05	N	<0,05	<0,05	<0,05	<0,05	>0,05	<0,05	>0,05
In	>0,05	<0,05	>0,05	<0,05	<0,05	<0,05	<0,05	In	<0,05	<0,05	<0,05	>0,05	<0,05	<0,05	>0,05
N+In	<0,05	<0,05	<0,05	>0,05	<0,05	<0,05	<0,05	N+In	>0,05	>0,05	<0,05	<0,05	<0,05	<0,05	>0,05

**CAT**	C							**POD**	C						
	2 h	7 h	10 h	24 h	72 h	144 h	240 h		2 h	7 h	10 h	24 h	72 h	144 h	240 h
N	>0,05	>0,05	>0,05	>0,05	>0,05	>0,05	>0,05	N	<0,05	<0,05	<0,05	<0,05	>0,05	<0,05	>0,05
In	>0,05	>0,05	<0,05	>0,05	>0,05	<0,05	>0,05	In	<0,05	<0,05	>0,05	<0,05	<0,05	<0,05	>0,05
N+In	>0,05	>0,05	>0,05	>0,05	<0,05	>0,05	>0,05	N+In	<0,05	<0,05	>0,05	>0,05	>0,05	<0,05	>0,05
